# Webinars about child and adolescent psychiatry can support professionals in war-affected regions: lessons from the ESCAP-Ukraine webinar initiative

**DOI:** 10.1007/s00787-026-03157-6

**Published:** 2026-08-04

**Authors:** Joerg M. Fegert, Emily Sitarski, Andrea Danese, Simone Toepfer, Elisa Pfeiffer, Elisa König, Ekaterina Mörtl, Michael Drechsler, Karen Schlaegel, Oksana Zbitnieva, Kateryna Bulavinova

**Affiliations:** 1 Department of Child and Adolescent Psychiatry, Psychosomatics and Psychotherapy, Ulm University, Steinhövelstrasse 5, Ulm, 89073 Germany; 2 European Society for Child and Adolescent Psychiatry, Kroonlaan 20, Brussel, 1050 Belgium; 3 Partner Site Ulm, Competence Center Public Child Mental Health, Steinhövelstraße 5, Ulm, 89075 Germany; 4 Partner Site Ulm, German Center for Mental Health (DZPG), Steinhövelstraße 5, Ulm, 89075 Germany; 5 Social, Genetic and Developmental Psychiatry Centre, Institute of Psychiatry, Psychology and Neuroscience, King’s College London, London, UK; 6 Department of Child and Adolescent Psychiatry, Institute of Psychiatry, Psychology and Neuroscience, King’s College London, London, UK; 7 National and Specialist CAMHS Clinic for Trauma, Anxiety, and Depression, South London and Maudsley NHS Foundation Trust, London, UK; 8 SOS-Kinderdörfer Weltweit, Ridlerstr. 55, München, 80339 Germany; 9 Department of Clinical Psychology and Child and Adolescent Psychotherapy, Catholic University Eichstätt-Ingolstadt, Levelingstraße 7, Ingolstadt, 85049 Germany; 10 Krisenchat gGmbH, Oranienstr. 6, Berlin, 10997 Germany; 11 Institute of Psychology, Universität Greifswald, Franz-Mehring-Str. 47, Greifswald, 17489 Germany; 12 Deutsche Traumastiftung, Weinhof 7, Ulm, 89073 Germany; 13 All Ukrainian Mental Health Programme ❝How are you❞?, Coordination Centre for Mental Health of the Cabinet of Ministers of Ukraine, Kyiv, Ukraine; 14 UNICEF Ukraine, 9B Hrushevskoho Str, Kyiv, 01021 Ukraine

**Keywords:** War, Professional capacity building, Trauma care, Psychosocial support, Webinars, Child and adolescent psychiatry, Ukraine, ESCAP, International collaborations

## Abstract

War has a profound impact on child and adolescent mental health and service provision. This requires creative internationally supported response to strengthen professional capacity in trauma care, psychosocial support and evidence based clinical practice in child and adolescent psychiatry. In September 2024, the European Society for Child and Adolescent Psychiatry (ESCAP)–Ukraine webinar initiative was launched in collaboration with Ukrainian governmental bodies, UNICEF, and international partners. Across two webinar series (2024–2026), the program delivered structured, expert-led online training to a wide multidisciplinary audience, including healthcare providers, educators, and social sector professionals. A total of 10,489 unique participants from all regions of Ukraine were reached, including frontline areas. Descriptive data highlight broad sectoral engagement, with the largest representation from healthcare (48.5%) and education (27.6%). The webinars, which combined live sessions, simultaneous translation, and interactive discussions, have become a critical and often accessible source of up-to-date professional education under wartime conditions. The initiative supports ongoing systemic reforms in Ukraine, particularly the integration of child and adolescent psychiatry into paediatric healthcare structures and the development of community-based mental health services. Complementary projects, including trauma-focused cognitive behavioural therapy (TF-CBT) training, e-learning programs, and international collaborations, further reinforce capacity building and service development. Overall, the ESCAP-Ukraine webinar series represents a scalable, cost-effective model for workforce development, knowledge exchange, and implementation of standardized, evidence-based mental health care in crisis settings which can serve as good practice examples for other regions. Continued international support and local ownership are essential for sustaining these efforts and addressing long-term mental health needs.

## Background and the need for innovation

War hits children first. Regardless of where it takes place, war has a profound impact on child and adolescent mental health and service provision. This requires creative internationally supported response to strengthen professional capacity in trauma-care, psychosocial support and evidence-based clinical practice in child and adolescent psychiatry.

Following the escalating situation in Ukraine and just before the start of Russian’s war of aggression on Ukraine, the Board of the European Society for Child and Adolescent Psychiatry (ESCAP) issued the statement “War hits children first” and thereby took a clear stance and offered support and guidance [1]. On the occasion of the International Children’s Day on 1 June 2023, President of the European Commission, Dr. Ursula von der Leyen, and President of Ukraine, Volodymyr Zelenskyy, issued a joint statement, too [2]. As part of the statement, they pledged to establish a specialised e-learning program for paediatricians and primary care medical staff with the objective to equip healthcare professionals with the necessary skills to provide trauma care and improve children’s mental health conditions.

## Goal: ESCAP-Ukraine webinars on fundamental topics of child and adolescent psychiatry

A medical conference on mental health, psychosocial support, and rehabilitation in Ukraine was held in Berlin on 1–2 February 2024, focusing on trauma care and the prevention of long-term consequences. Participation in the conference provided an opportunity to engage with key stakeholders from Ukraine’s mental health sector, governmental institutions, and international organizations [3]. These discussions led to the development of the idea for a webinar series.

## Phases taken for development and implementation of the ESCAP-Ukraine webinars

The proposal was presented to the ESCAP Board for approval, where it received strong endorsement. The German Trauma Foundation was approached for support, and then leading experts from across Europe were invited to express their willingness to contribute to this webinar series.

Thanks to the local support of UNICEF Ukraine, in September 2024, ESCAP was able to start the first series of webinars, which provided Ukrainian professionals with up-to-date knowledge on trauma therapy, psychosocial first aid and resilience building. The project was implemented within the framework of the All-Ukrainian Mental Health Programme ‘How are you?’, an initiative of Ukraine’s First Lady, with the support of the ESCAP office and the German Trauma Foundation in cooperation with the Coordination Centre for Mental Health of the Cabinet of Ministers of Ukraine, the Ministry of Health of Ukraine, the National Health Service of Ukraine (NHSU), UNICEF Ukraine. International experts were volunteering their time and presented on a variety of topics in webinars hosted on Zoom and streamed via YouTube and Facebook. The presentations were simultaneously translated into Ukrainian and were followed by an interactive 30 minutes of Q&A. The translated slides were also made available to the participants after the session and the videos are available on YouTube in both English and Ukrainian. The webinars were accredited by the Baden-Württemberg Medical Association. Participants received a certificate of attendance upon completion which in turn, were recognised in Ukraine as professional development. 13 webinars took place in the first series in 2024/2025 and 12 in the second series in 2025/2026.

For the first time in 2025, the International Charlemagne Prize came with prize money. To ESCAP’s surprise and delight, the 2025 Charlemagne Prize winner, Dr. Ursula von der Leyen, and the Charlemagne Prize Foundation awarded a substantial part of this prize money to the German Trauma Foundation in order to support two projects related to ESCAP’s activities. This enabled ESCAP to run a second series of the webinars and the third series is, despite the continuing challenging circumstances in Ukraine, in planning.

## Outcomes of the ESCAP-Ukraine webinars: scale and reach

From September 2024 to December 2025 the webinars reached 10,489 unique participants. These came from 24 regions of Ukraine and the city of Kyiv, including 2,402 settlements.

The largest number of participants came from Kyiv City, Kyiv region, Kharkiv, Dnipropetrovsk, and Odesa regions. It is also notable that the series was watched in frontline regions and areas most affected by shelling and blackouts, including Kharkiv, Dnipropetrovsk, Odesa, Sumy, Chernihiv, Kherson, and Donetsk regions, as well as Kyiv and Kyiv region. There were also participants from 21 other countries and 92.6% of participants were women. This reflects the actual workforce structure of the healthcare, education, and social sectors in Ukraine, where the majority of professionals are women [4].

New participants continued to join the series: of the 3,104 participants who registered in January-February 2026, one third were new participants. Typically, 600 to 4,000 people registered for each event. From start to finish, between 229 and 699 participants usually attended the webinars live on Zoom.

### Participants: sectoral representation

The sample comprised 10,489 participants across multiple sectors. The largest proportion was drawn from the healthcare sector (48.53%, *n* = 5,090), followed by the education sector (27.64%, *n* = 2,899). Smaller shares of participants represented the civil society sector (10.23%, *n* = 1,073) and the social sector (4.56%, *n* = 478).

A limited number of respondents were affiliated with the military, veterans, or the State Security Service (0.89%, *n* = 93), as well as the law enforcement and justice sector (0.98%, *n* = 103), including police, the State Emergency Service (DSNS), border guards, courts, the Ministry of Justice, and the Prosecutor’s Office.

The remaining participants (7.18%, *n* = 753) were categorized as “other”, encompassing individuals from the business sector, unemployed persons, individuals on parental leave, as well as representatives from Energoatom, the Office of the President, various ministries, Parliament, local authorities, and the media.

### Participants: healthcare professionals

Among the 5,090 participants from the healthcare sector, the largest subgroup consisted of other medical professionals (29.37%, *n* = 1,495), followed by nurses and midwives (20.1%, *n* = 1,022). Paediatricians and family doctors accounted for 15.52% (*n* = 790), while heads of healthcare facilities and department heads represented 11.4% (*n* = 580).

Psychiatrists and psychotherapists comprised 11.1% of the sample (*n* = 565), including 188 child psychiatrists (3.7%). Psychologists and clinical psychologists accounted for 8.23% (*n* = 419). Smaller proportions included medical interns (2.8%, *n* = 143) and neurologists (1.47%, *n* = 75), among whom 37 specialized in paediatric neurology.

### Participants: medical universities, colleges^1^

In addition, 205 participants were affiliated with medical universities, including 154 professors, 43 students, and 8 medical interns. A further 53 participants represented medical colleges, of whom 48 were professors and 5 were students.

The webinars were also attended by parents of children with mental health and neurodevelopmental disorders, who frequently raised questions related to their specific cases.

### Participants: education sector

Participants from the education sector (*n* = 2,899; excluding medical universities and colleges) comprised a diverse group of professionals. Psychologists represented the largest subgroup, accounting for more than one third of all education sector participants. Additional participants included staff from general secondary schools, preschools, and lyceums, as well as representatives of inclusive resource centres. The sample also encompassed university professors and members affiliated with the National Academy of Sciences of Ukraine.

Across all professional sectors, a total of 3,573 psychologists participated in the webinar series.

### Participants: social sector

Participants from the social sector (*n* = 478) included representatives from a wide range of institutions and services. These comprised social service centres, resilience centres, child protection services, and rehabilitation centres. The sample also included representatives from family-type children’s homes, the pension fund, and shelters for victims of violence.

## Discussion

The reach and the impact of the webinars so far have been immense. The active participation, even under extreme circumstances such as air raids and power cuts, are impressive. This series has effectively become the only accessible source of up-to-date, evidence-based knowledge in their field for Ukrainian professionals.

Notably, university faculties themselves regularly attend the webinars, using them as a resource for their own learning and teaching. This is particularly relevant in the context of ongoing reforms in the area of mental health in Ukraine, as initiated by Ukraine’s First Lady, and in particular in child and adolescent mental health services. Effective July 2025, a major policy shift introduced the integration of child and adolescent psychiatry services into regional children’s hospitals, replacing the previous model centred on adult oblast psychiatric hospitals^2^. This reform requires the development of new service delivery models aligned with international standards, as well as substantial capacity building for both clinical and managerial staff in children’s hospitals.

In parallel, Mental Health Centres providing specialised outpatient services are being established at oblast children’s hospitals in each region. These centres are expected to destigmatize and provide targeted services for children and adolescents. Their development likewise requires significant workforce training and capacity building. At the same time, more medical universities are beginning to introduce specializations in child and adolescent psychiatry. In this context, the webinar series is playing a crucial role as a platform for professional education and knowledge exchange.

However, the ESCAP-Ukraine webinar series does not stand alone. There are numerous additional initiatives in Ukraine that further reinforce capacity building and service development, some of which involve members of the ESCAP Board. We would like to highlight a selection of these initiatives here.

### Other support initiatives

#### Trauma-focused cognitive behavioural therapy: “TF-CBT Ukraine”

Researchers from the Department of Child and Adolescent Psychiatry, Psychosomatics and Psychotherapy at the Ulm University Hospital, set up the research and care project “TF-CBT Ukraine” (Trauma-focused cognitive behavioural therapy in Ukraine). Among the collaboration partners and funders are the TF-CBT treatment developers, certified international TF-CBT and EMDR trainers, the National Psychological Association of Ukraine, the National Child Traumatic Stress Network (USA), the Ministry of Health of Ukraine and Ministry of Education and Science of Ukraine and the “Mental health for Ukraine Project”, implemented by GFA Consulting Group GmbH, the Porticus Foundation, and the CARES Institute in the US. Since the project started in 2022, 10 training cohorts with 243 trained professionals and 67 TF-CBT therapists were certified. The project was then moved to collaboration partners in Ukraine who went on to train more than 100 Ukrainian therapists with several international partners. Moreover, an EMDR therapy programme (over 30 trained EMDR therapists, 150 children treated) was evaluated and extra courses on topics such as self-care for therapists, treatment of traumatic grief or adapting TF-CBT to ongoing war scenario were offered. Participation was open to mental health care providers from across Ukraine and those who had already fled the country due to the war.

The close contact between the project team and Ukrainian professionals gave rise to the idea for this in-depth professional exchange, which took place in Ulm in February 2026 and was also supported by the International Charlemagne Prize. The program of the visit included workshops and specialist lectures by the child and adolescent psychiatry team in Ulm, exchanges with various professional groups at the clinic, and a visit to the Clinic for Child and Adolescent Psychiatry in Böblingen, Germany, to learn about another German teaching clinic in the region. A meeting with the mayor of Ulm took place on 17 February. The project demonstrated that evidence-based trauma therapy works even in wartime conditions and that the collaboration between Ukrainian and international experts is crucial [5–9]. The demand for training programmes remains high and the project has a waiting list of more than 100 therapists. The evaluation showed that the levels of satisfaction with the programme among therapists and trainers was very high and so were the levels of adherence and acceptance among Ukrainian therapists [6]. Despite the war situation, dropout rates were low and recruitment rates high. Furthermore, a significant reduction in PTSD symptoms from pre- to post-treatment with large effect sizes was achieved [6, 7]. Several publications resulted from this project [5–9].

#### Development of e-learning course and manualized intervention under the EU programme ‘Trauma-informed approaches in Better Care’

Under the Government of Ukraine’s national Better Care reform, a partnership between Ulm University Hospital, V. N. Karazin Kharkiv National University and UNICEF, funded by the European Union, aims to strengthen and harmonize mental health and psychosocial support (MHPSS) services for children and caregivers, including those in alternative care. The programme focuses on developing a trauma-informed approach to care, enhancing professional competencies and promoting alternatives to institutional care. Using the Kharkiv region as a model, the initiative includes the creation of an e-learning course and a manualized intervention to support consistent, evidence-based MHPSS practices.

This project contributes by developing an e-learning course and a manualized intervention for Ukrainian psychology and social work students as well as professionals.

The e-learning course provides knowledge on trauma-related disorders, promotes a trauma-informed perspective, and introduces practical coping and self-care strategies for both young people and caregivers. A first cohort of 84 participants has completed the online course and have also provided feedback. Participants are very highly satisfied with the quality of the online course and also reported a high level of perceived competence gain. Based on the evaluation results, the course was being revised, e.g., video clips with role plays, case studies, or short quizzes were incorporated to make the content even more interactive.

The manualized intervention consists of three workbooks, including specific and techniques for emotion regulation and stabilization (based on the manual START (Stress-Traumasymptoms-Arousal-Regulation-Treatment) [10, 11]), sleep hygiene (based on the manual “Ready for Landing”, [12]), coping with grief or self-care, among others. The workbooks were made available to a subset of 24 course graduates, along with a one-day workshop in which the materials were explained. These participants “tested” the workbook content with children, adolescents, and families during an implementation phase. The workbooks were evaluated using a combination of online surveys and one-on-one interviews. The findings indicate that the materials effectively enhance practitioners’ confidence and competence in the thematic areas covered by the workbooks, such as emotional regulation and sleep hygiene. The quality of the content and the creative exercises were highly praised for their practical relevance and usability. Based on this feedback, the workbooks were revised, particularly with regard to the design of the printed versions and their cultural relevance. Currently, the project funder is exploring different options for the sustainable implementation of the project outputs, including the e-learning course and the workbooks.

#### SOS Children’s Villages worldwide

Across Ukraine, SOS Children’s Villages are providing critical support to children and families displaced or affected by the conflict. Before the escalation in 2022, SOS Children’s Villages were active in the Kyiv and Luhansk regions. They are now active in the Kyiv region, as well as in Ivano-Frankivsk, Poltava, and Mycolaiv. In addition to humanitarian aid, their activities focus on programs for family support and foster care assistance. Around 166,000 children and parents have received emergency supplies, counselling, and psychosocial assistance through the organization’s social centres. In over 120 child protection centres, children have safe spaces to learn, play, and grow [13].

More than 185,000 children and parents have benefited from psychological support, including via mobile teams reaching remote or conflict-affected areas. Holiday camps give children the chance to relax and enjoy a safe, nurturing environment [13].

965 children injured in the war and their families are receiving medical care, psychological support, and rehabilitation [13]. SOS Children’s Villages also promote long-term care for war orphans through foster families. Over 67,000 people have received financial assistance, and more than 1,200 individuals have been supported with training for career reorientation.

During the winter, essential supplies such as firewood, heaters, and winter clothing are distributed. Families also receive help with home insulation and repairs, access to safe and warm spaces, and hot meals [13].

Beyond Ukraine, SOS Children’s Villages support Ukrainian families in eight European countries, providing accommodation, psychosocial care, education, and integration assistance, including in Poland, Romania, Hungary, Italy, Austria, and Germany [13].

#### Collaboration King’s College London – UNICEF Ukraine

ESCAP is also partnering in a programme to enhance specialist child and adolescent mental health services in Ukraine. The programme is funded by UNICEF Ukraine and led by researchers at King’s College London in collaboration with the Ukrainian National Mental Health Coordination Centre. The programme aims to develop recommendations for service delivery models, a specialist training curriculum, and the curriculum for a clinical training workshop in child and adolescent psychiatry.

#### Krisenchat (crisis chat) Ukrainian

krisenchat Ukrainian [14] is a free, chat-based crisis counselling service available to people of all ages. The focus of the project is providing fast, confidential, and professional help in Ukrainian and Russian languages to all the people affected by the war in Ukraine. Having started on March 1, 2022, krisenchat Ukrainian already conducted over 56,000 consultations via chat with almost 27,000 people. The median waiting time for the psychological consultation to start is 7 minutes. All the staff counsellors are professional psychologists who have also undergone intensive internal training in chat counselling, krisenchat’s volunteers are also professionals and the interns counsel under supervision. The chat approach focuses on stabilisation, emotional co-regulation, and further referral when necessary.

What is especially noteworthy is that, for several projects, the central organization and their continued development are now being carried forward from within the country itself—despite the ongoing realities of war. This shift not only reflects remarkable adaptability, but also a profound sense of ownership and determination.

## Conclusion

The webinar series has become a rapid mechanism for strengthening the system, an effective and cost-efficient tool for workforce development and a platform for establishing unified evidence-based standards. The initiative combines scale, quality, international expertise, and systemic impact on the reform of child and adolescent psychiatric care.

After four years, there is a real risk that the situation fades into the background of our awareness. This sense of normalization should be consciously resisted. Attention, solidarity, and support remain not only necessary, but vital and as such the webinar series must absolutely continue.

Moreover, it has helped raise awareness of other initiatives and foster a stronger collaboration between them. The joint organisers of the webinars and the authors of this paper believe it is important to connect these initiatives and to establish as many externally supported efforts as possible on a sustainable basis within Ukraine. This ranges from the training of supervisors and instructors in Ukraine to e-learning programs developed together with Ukrainian web designers, ensuring that the work does not remain dependent on external support but becomes a lasting implementation in Ukraine - an effort that will, unfortunately, continue to deal with the consequences of the ongoing and highly stressful war for many years to come. Approaches and lessons learned from these initiatives can be used as a good practice example for other areas affected by war.

## Data Availability

No datasets were generated or analysed during the current study.

## Footnotes

1 In Ukraine, medical universities are separate specializedinstitutions and are not part of comprehensive (academic) universities. Theyoperate as independent higher education institutions focused exclusively onmedical and health sciences training, typically under the oversight of theMinistry of Health.

2 Oblast psychiatric hospitals are regional psychiatric hospitals serving administrative regions (“oblasts”) in several Eastern European countries.
